# *FOXP3* Allelic Variants and Haplotype Structures Are Associated with Aggressive Breast Cancer Subtypes

**DOI:** 10.1155/2017/6359603

**Published:** 2017-06-21

**Authors:** Bruna Karina Banin Hirata, Roberta Losi Guembarovski, Glauco Akelinghton Freire Vitiello, Alda Losi Guembarovski, Karen Brajão de Oliveira, Maria Angelica Ehara Watanabe

**Affiliations:** ^1^Department of Pathological Sciences, Biological Sciences Center, State University of Londrina, Celso Garcia Cid Highway, Pr 445 Km 380, Campus Universitário, 86057-970 Londrina, PR, Brazil; ^2^Department of General Biology, Biological Sciences Center, State University of Londrina, Celso Garcia Cid Highway, Pr 445 Km 380, Campus Universitário, 86057-970 Londrina, PR, Brazil; ^3^Department of Pathology, Clinical and Toxicological Analysis, Health Science Center, State University of Londrina, Celso Garcia Cid Highway, Pr 445 Km 380, Campus Universitário, 86057-970 Londrina, PR, Brazil

## Abstract

*FOXP3* genetic polymorphisms have been associated with cancer development and prognosis. In this context, the present study aimed to evaluate the g.10403A>G (rs2232365) polymorphisms and g.8048A>C (rs3761548), in aggressive breast cancer (BC) subtypes, including, Luminal B HER2+ (LB), HER2-enriched (HER2+), and triple-negative (TN). Polymerase chain reaction followed by enzymatic restriction was performed to genotyping 117 BC samples and 300 controls. A significant association of AA genotype (g.10403A>G) in relation to BC susceptibility (OR = 1.93; 95% CI = 1.01–3.66; *p* = 0.046) was observed. The GG (g.10403A>G) genotype was correlated with higher proliferation index (Ki-67) in HER2+ subtype (*τ* = 0.47; *p* = 0.019) and advanced TNM staging in TN (*τ* = 0.23; *p* = 0.032). A correlation of AA genotype (g.8048A>C) with higher Ki-67 (*τ* = −0.47; *p* = 0.018) and lower histological grade (*τ* = 0.39; *p* = 0.026) in HER2+ was also found. GA haplotype was correlated with lower histological grade (*τ* = −0.15; *p* = 0.009) and higher Ki-67 (*τ* = 0.43; *p* = 0.036) in HER2+ and advanced staging in TN (*τ* = 0.29; *p* = 0.044). On the other hand, AC haplotype was correlated with lower Ki-67 (*τ* = −0.54; *p* = 0.005) and staging (*τ* = −0.29; *p* = 0.027) in HER2+ and TN respectively. Results showed that *FOXP3* influence regarding clinical outcome depends greatly on the BC subtype and indicated this transcription factor as a promising marker in aggressive BC subtypes.

## 1. Introduction

The National Cancer Institute (INCA) estimated 57,960 new cases of breast cancer (BC) for 2016 and 2017 in Brazil. It is worth noting that, regardless of nonmelanoma skin cancer, the mammary tumor is the most common among women in many regions of the country, accounting for high morbidity and mortality [1].

BC represents a complex and heterogeneous disease that comprises distinct pathologies, histological features, and clinical outcome. The status of estrogen receptor (ER), progesterone receptor (PR), human epidermal growth factor receptor type 2 (HER2), and proliferation index Ki-67 has been used as predictive markers to identify high-risk phenotypes and for selection of most efficient therapies [2–4].

These molecular markers are also generally used to define BC subtypes, namely Luminal A (LA; ER/PR^+^HER2^−^), Luminal B (LB; ER/PR^+^HER2^+^ or ER/PR^+^HER2^−^Ki-67^High^), HER2-enriched (HER2+; ER/PR^−^HER2^+^), and basal-like, also termed as triple-negative (TN; ER/PR^−^HER2^−^) [5]. Among these subtypes, the basal-like has the worst prognosis, while Luminal A has the best [6]. Within tumors that present HER2 overexpression, Luminal B (hormonal receptors positive) was associated with better prognosis compared with the HER2-enriched subtype [7].

Forkhead box P3 (FOXP3) is an essential transcription factor to the development and functions of Regulatory T cells (Tregs) [8]. Increased levels of FOXP3^+^Tregs in peripheral blood and tumor microenvironment have been reported in diverse cancer types, including the breast one [9]. These cells play an important role in immune response suppression and thus may contribute to tumorigenesis.

The accumulation of Tregs in local lymph nodes or in tumors is associated with an unfavorable prognosis [10, 11]. Although Tregs are the major cell type expressing FOXP3, it has been demonstrated that tumor cells themselves can express this protein, such as those in pancreatic cancer [12], melanoma [13], and breast tumors [14, 15]. Moreover, FOXP3 expression in tumor cells could be an independent strong prognostic factor for distant metastasis in BC [16]. However, in contrast with this data, this transcription factor was also shown to be a tumor suppressor gene, acting as a transcriptional repressor of *SKP2* and *HER2*, two important BC oncogenes [17, 18].

Considering FOXP3 dual role in tumor microenvironment, investigation of polymorphisms and their possible associations with cancer may shed light on the molecular cancer pathogenesis and open new perspectives to susceptible individual screening [19].

Polymorphisms in the *FOXP3* gene may change its product quantitatively or functionally, thereby contributing to an immune imbalance in cancer. To date, *FOXP3* allelic variants have been associated with a variety of immune-related diseases, such as allergic rhinitis [20], idiopathic infertility, and endometriosis-related infertility [21]. Furthermore, *FOXP3* polymorphisms have also been associated with different types of cancer, such as Wilm's tumor [22], hepatocellular carcinoma [23], colorectal cancer [24], and nonsmall cell lung carcinoma [25]. However, few studies have investigated BC patients [26, 27], especially in their molecular subtypes and in relation to their clinical outcomes.

In this context, the present study aimed to investigate possible association between two *FOXP3* single nucleotide polymorphisms (SNPs) regarding susceptibility and clinical outcome in aggressive BC subtypes (LB, HER2+, and TN) from a South Brazilian sample.

## 2. Materials and Methods

### 2.1. Ethical Aspects and Sample Characterization

Patients and controls were informed in detail regarding the research, and the consent term was obtained. In the present study, 107 peripheral blood samples (5 mL) collected with EDTA as anticoagulant and 10 paraffin-embedded tissues from patients attended in the Cancer Hospital of Londrina, Londrina, Paraná, Brazil (CHL) were included. In total, 117 BC samples were obtained, of which 37 were diagnosed as Luminal B HER2+ (LB), 26 as HER2-enriched (HER2+), and 54 as triple-negative (TN) subtypes.

For the control group, 300 blood samples were collected from women of same geographic region, without BC, proved by clinical and imaging examination, no self-declared BC family history or personal history of any malignant disease.

Clinicopathologic parameters data and immunohistochemical classification of BC subgroups were retrieved from patients' medical register available at CHL. Prognostic parameters included tumor size, lymph node commitment, proliferation index Ki-67, histological grade, and clinicopathological staging (Tumor/Node/Metastasis classification), which were determined according to the Union of International Control of Cancer classification criteria [28].

### 2.2. Genomic DNA Extraction

Genomic DNA was obtained from peripheral blood cells using Biopur Mini Spin Plus Kit (Biometrix Diagnostica, Curitiba, Brazil), according to the manufacturer's instructions. From the formalin fixed and paraffin-embedded samples, DNA was extracted using innuPREP DNA Mini (Analytik Jena, Jena, Germany), according to manufacturer's protocol. All samples were quantified by NanoDrop 2000c®Spectrophotometer (Thermo Scientific, Wilmington, USA) at a wavelength of 260/280 nm, and the final preparations were stored at −20°C.

### 2.3. FOXP3 Genotyping

Polymerase chain reaction (PCR) followed by enzymatic restriction (PCR-RLFP) was performed to genotype rs2232365 and rs3761548 SNPs (HGVS names: g.10403A>G and g.8048AC, resp., according to Gen Bank Accession number NG_007392.1).

For g.10403A>G genotyping, the following primers were used: 5′-AGGAGAAGGAGTGGGCATTT–3′ (forward) and 5′-TGTGAGTGGAGGAGCTGAGG–3′ (reverse), according to Paradowska-Gorycka, Jurkowska [29]. The g.8048A>C genotyping was performed with the following primers: 5′-GGCAGAGTTGAAATCCAAGC–3′ (forward) and 5′-CAACGTGTGAGAAGGCAGAA–3′ (reverse), according to He et al. [25]. The PCR was conducted using 1X of PCR Buffer (20 mM of Tris-HCl ph 8.5; 50 mM of KCl), 0.8 mM of MgCl_2_, 0.1 mM of dNTP, 0.2 *μ*M of each primer, 0.05 U/*μ*L of Taq DNA polymerase, and 4 ng/*μ*L of genomic DNA diluted in ultra-pure H_2_O (Milli-Q) to complete a final volume of 25 *μ*L per reaction tube. Negative controls were employed to make sure that no contaminants were introduced. The cycling protocol, used to both *FOXP3* polymorphisms, was a denaturation at 94°C for 5 min, 35 cycles of 45 sec at 94°C, 45 sec at 59°C to g.10403A>G or 65°C to g.8048A>C, 45 sec at 72°C, and 10 min of final elongation at 72°C. PCR products (5 *μ*L) of g.10403A>G, with 249 bp, were digested overnight at 55°C with 1 unit/reaction of BsmBI restriction endonuclease (New England Biolabs, Beverly, USA), generating two fragments of 132 bp and 117 bp corresponding to allele G. The PCR products (6 *μ*L) of g.8048A>C, with 155 bp, were digested overnight at 37°C with 2 units/reaction of PstI restriction endonuclease (New England Biolabs, Beverly, USA), generating two fragments of 80 bp and 75 bp that correspond to allele C. All PCR and digested products were analyzed on polyacrylamide gel (10%), stained with silver nitrate.

### 2.4. Haplotype Analysis

*FOXP3* haplotypes were determined based on the genotypes of all study participants using PHASE software version 2.1.1 [30, 31]. Permutation test was also performed, using the same software, to check for haplotype distribution differences among controls and BC subgroups.

### 2.5. Statistical Analysis

Binary logistic regression analyses were conducted to investigate possible associations between polymorphisms or haplotype structures and BC, controlled by age. Associations were tested considering genotypic models (heterozygotes or variant homozygotes versus wild homozygotes), dominant model (heterozygotes and variant homozygotes versus wild homozygotes), and recessive model (variant homozygotes versus wild homozygotes and heterozygotes). In the association study of *FOXP3* haplotypes, the following models were analyzed: AC dominant (AA, GC, and GA carriers versus AC carriers), AC recessive (AA, GC, and GA carriers versus ACAC), AA dominant (AC, GC, and GA carriers versus AA carriers), GC dominant (AC, AA, and GA carriers versus GC carriers), GC recessive (AC, AA, and GA carriers versus GCGC), GA dominant (AC, AA, and GC carriers versus GA carriers), and GA recessive (AC, AA, and GC carriers versus GAGA). The AA dominant model was not analyzed in TN subtype because the group did not present this haplotype.

Correlations between polymorphisms or haplotype structures and clinical parameters were assessed by Kendall's tau-b rank correlation coefficient.

All statistical analyses were performed in software SPSS 22.0 version (SPSS Inc., Chicago, USA) and were two-tailed, with 5% significance level.

## 3. Results

In the present study, the median age of BC patients was 51 (±14) years and of control group was 55 (±13) years (*p* = 0.118). The prognostic parameters in general BC patients and in different subtypes are shown in Table 1. Some parameters were not available.

**Figure 1:**
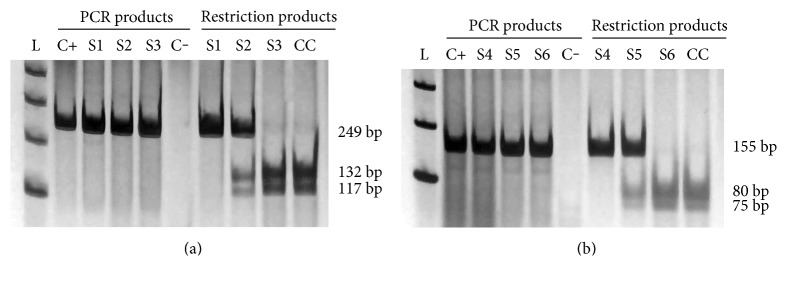
Eletrophoretic profiles of *FOXP3* polymorphisms. (a) Eletrophoretic profiles of g.10403A>G (rs2232365). (b) Eletrophoretic profiles of g.8048A>C (rs3761548). L: Ladder 100 bp; C+: positive control; C−: negative control; CC: cleavage control; S1: homozygote genotype AA; S2: heterozygote genotype AG; S3: homozygote genotype GG; S4: homozygote genotype AA; S5: heterozygote genotype AC; S6: homozygote genotype CC.

Eletrophoretic profiles of *FOXP3* polymorphisms are shown in Figure 1. Genotype distribution, allele, and haplotype frequencies for both polymorphisms are showed in Table 2. The minor allele frequency (MAF) of g.10403A>G and g.8048A>C was consistent with the corresponding frequencies reported in 1000 Genomes project (https://www.ncbi.nlm.nih.gov/variation/tools/1000genomes/).

In relation to FOXP3 haplotypes, the predominant was the AC, both in controls and in all BC subgroups, while the less common was the AA. The haplotype frequencies from controls were compared with African, European, American, and Asian populations, using the publicly available data from the 1000 genome project obtained through web-based application LDlink [32]. The haplotype frequencies were significantly different from these populations (*p* < 0.05 by *χ*^2^ test). No significant difference was found in haplotype distribution between controls and BC patients in the general sample (*p* = 0.52).

In the present study, in the total sample, AA genotype of g.10403A>G was associated with BC susceptibility (OR = 1.93; 95% CI = 1.01–3.66; *p* = 0.046). No association was found to dominant (GG versus (AG + AA)) or recessive ((GG + AG) versus AA) models. Also, no association with BC susceptibility was found to g.8048A>C polymorphism, in genotype, dominant, or recessive models.

Therefore, no significant association between haplotypes and BC susceptibility was found, both in total sample or in different subtypes. Although, a strong tendency of association of AC haplotype with BC protection, in total sample in the recessive model (OR = 0.58; 95% CI = 0.33–1.01; *p* = 0.053), and TN subtype in dominant model (OR = 0.55; 95% CI = 0.28–1.07; *p* = 0.08) was observed. Also, a tendency was found between AA haplotype, in dominant model, and BC protection in LB subtype (OR = 0.19; 95% CI = 0.03–1.05; *p* = 0.06).

The analysis considering clinical parameters showed a significant correlation between GG genotype of g.10403A>G polymorphism and higher proliferation index Ki-67 in HER2 + subtype (*τ* = 0.47; *p* = 0.019) and advanced TNM staging in TN subtype (*τ* = 0.23; *p* = 0.032). A significant correlation of AA genotype of g.8048A>C polymorphism with higher Ki-67 (*τ* = −0.47; *p* = 0.018) and lower histological grade, in HER2+ subtype (*τ* = 0.39; *p* = 0.026) (Table 3), was also found.

Furthermore, a significant correlation of GA haplotype with lower histological grade (*τ* = −0.15; *p* = 0.009) and higher Ki-67 (*τ* = 0.43; *p* = 0.036) in HER2+ subtype and with advanced staging in TN (*τ* = 0.29; *p* = 0.044) was found. The AC haplotype was correlated with lower Ki-67 (*τ* = −0.54; *p* = 0.005) and TNM staging (*τ* = −0.29; *p* = 0.027) in HER2+ and TN subtypes, respectively (Table 4).

## 4. Discussion

In the present study, *FOXP3* g.10403A>G and g.8048A>C polymorphisms were analyzed in 117 BC patients and 300 neoplasia-free controls. Present results indicated a significant association of AA homozygous genotype (g.10403A>G) in relation to BC susceptibility (OR = 1.93, 95% CI = 1.01 to 3.66), suggesting that individuals who had inherited both copies of the allelic variant are more susceptible for BC development than wild homozygous (GG) individuals.

To our knowledge, there are no studies reporting any significant association between g.10403A>G and BC susceptibility, but significant associations have been proposed with other diseases, such as psoriasis vulgaris (MAF: allele G, cases = 0.19; controls = 0.27) [33], vitiligo (MAF: allele G, cases = 0.34; controls = 0.28) [34], unexplained recurrent spontaneous abortion (MAF: allele A, cases = 0.29; controls = 0.40) [35], idiopathic recurrent pregnancy loss (MAF: allele G, cases = 0.40; controls = 0.29) [36], and autism spectrum disorders (MAF: allele A, cases = 0.09; controls = 0.12) [37].

Wu et al. [38] performed an extensive search for transcriptional factor-binding sites and found that g.10403A>G SNP is located in a putative binding site for the transcription factor GATA-3. More importantly, only when the allele A exists, this transcription factor can bind the promoter region of *FOXP3*. According to Wang et al. [39], defective function of both GATA-3 and FOXP3 itself led to ablation of Treg cells, suggesting that the combined function of these genes is essential for FOXP3 expression, highlighting the indispensable role of GATA-3 in regulating Treg cell function. In this context, the allele A of g.10403A>G may be associated with increased FOXP3 expression and, consequently, in the maintenance of Treg function, contributing to suppression of antitumor immune response. This fact may explain the positive association between this polymorphism and increased BC susceptibility.

In the present study, no association was found between g.8048A>C and BC susceptibility, neither in general sample nor in different subtypes. Similar observations were made by Raskin et al. [40] in Israeli population (MAF: allele A, cases = 0.47; controls = 0.47), Zheng et al. [41] in Han Chinese population (MAF: allele A, cases = 0.19; controls = 0.18), and Jahan et al. [26] in Indian population (MAF: allele C, cases = 0.47; controls = 0.44). Additionally, a meta-analysis performed by Jiang and Ruan [27] (MAF: allele A, cases = 0.37; controls = 0.34) indicated that g.8048A>C is not associated with BC, but with susceptibility to hepatocellular carcinoma and nonsmall cell lung cancer.

No significant association between different *FOXP3* haplotypes and BC susceptibility was observed, either in the general BC sample or in the different subtypes. To date, there are no studies relating the g.10403A>G and g.8048A>C haplotypes to BC susceptibility or clinical outcome, emphasizing that the present study is the first in the literature to describe this lack of association.

In addition, regarding BC prognosis, the present results showed a significant correlation of GG genotype (g.10403A>G) with higher proliferation index Ki-67 in HER2+ subtype and advanced TNM staging in TN subtype. To date, this is the first study that observed a correlation between g.10403A>G polymorphism and BC prognostic parameters.

As previously discussed, the allele G may be related to lower expression of FOXP3 due the lost binding site to GATA-3. Many studies have shown that, in BC, *FOXP3* could be considered a tumor suppressor gene, conferring a better prognosis [42, 43].

Despite *FOXP3*, g.8048 A>C may not be playing a role in BC susceptibility in Brazilian women; we report a significant correlation of AA genotype with higher Ki-67 and lower histological grade in HER2+ subtype. No correlation of this polymorphism with prognostic parameters was found in TN subtype, which is in accordance with a previous study developed by our research group [44].

Like the GG genotype of g.10403A>G, the AA of g.8048A>C also appears to be related to *FOXP3* lower expression. Shen et al. [45] observed that psoriatic patients with this genotype have reduced *FOXP3* expression. These authors demonstrated that the C to A change causes binding loss to E47 and c-Myb transcription factors, leading to a defective *FOXP3* gene transcription.

Furthermore, Jahan et al. [46] observed a highly significant association of AA (g.8048A>C) with BC advanced stages (III and IV). In the present study, no correlation with tumor stage was found and, perhaps, this discrepant result is due to the BC subtypes studied. These authors did not stratify the BC sample and, probably, included subtypes of better prognosis, such as Luminal A and Luminal B HER2-, unlike the present study, which comprised only more aggressive BC subtypes.

In contrast, we found correlation of AA genotype (g.8048A>C) with higher histological grade. Ohara et al. [47] analyzed *FOXP3* expression in breast tumor by qRT-PCR and observed a significant correlation with higher histological grade. These authors attributed the correlation with worse prognosis to Treg infiltration. Other study using immunohistochemistry technique also showed association of FOXP3 expression by tumor cells with higher histological grade [48]. However, in this study, all tumor samples showed cytoplasmic or both cytoplasmic and nuclear FOXP3 expression, suggesting frequent deregulation of FOXP3 localization and failure to translocate to the nucleus in breast cancer cells and explaining the correlation with worse prognosis. In this context, the positive correlation of g.8048A>C with histological grade may reflect the lower functional Treg infiltration in tumor bed.

Furthermore, significant correlations between *FOXP3* haplotypes and prognostic parameters were found. The present study showed a correlation of AC with better prognosis, such as lower proliferation index and staging, in HER2+ and TN, respectively. As discussed above, the polymorphisms may affect the expression of gene, and, in this context, AC haplotype may be related with higher FOXP3 expression, possibly explaining the correlation with better prognosis, since this transcription factor is considered a BC tumor suppressor gene.

In conclusion, the present study showed, for the first time, a significant association of *FOXP3* g.10403A>G with susceptibility and prognosis of aggressive BC. Although the g.8048A>C may not be associated with BC susceptibility, significant correlations with clinical outcome were found. Furthermore, present study also showed, for the first time, different correlations regarding prognosis in LB, HER2+, and TN, highlighting that the influence of allelic variants may depend on tumor subtype. Moreover, the dual role of *FOXP3*, participating in Treg cell development and function from one side and acting as a tumor modulator gene from other side should not be ignored.

## Figures and Tables

**Table 1:** Prognostic parameters in total BC sample and in aggressive subtypes.

Prognostic parameters		Total BC	LB	HER2+	TN
Tumor size	<1.5 cm	10 (8.9%)	3 (8.3%)	2 (8.7%)	5 (9.4%)
	1.5–3.0 cm	57 (50.9%)	23 (63.9%)	14 (60.9%)	20 (37.8%)
	>3.0 cm	45 (40.2%)	10 (27.8%)	7 (30.4%)	28 (52.8%)

TNM staging	I	19 (18.1%)	7 (20%)	5 (19.2%)	7 (15.9%)
	II	39 (37.2%)	14 (40%)	8 (30.8%)	17 (38.6%)
	III	37 (35.2%)	12 (34.3%)	9 (34.6%)	16 (36.4%)
	IV	10 (9.5%)	2 (5.7%)	4 (15.4%)	4 (9.1%)

Histological grade	II	30 (26.8%)	12 (33.3%)	8 (34.8%)	10 (18.9%)
	III	82 (73.2%)	24 (66.7%)	15 (65.2%)	43 (81.1%)

Ki-67	Low	7 (8.8%)	4 (19.0%)	0 (0.0%)	3 (7.3%)
	Moderate	25 (31.2%)	9 (42.9%)	7 (46.7%)	9 (23.6%)
	High	48 (60.0%)	8 (38.1%)	8 (53.3%)	32 (69.1%)

Lymph nodes commitment	No	54 (49.1%)	19 (52.8%)	12 (52.2%)	23 (45.1%)
	Yes	56 (50.9%)	17 (47.2%)	11 (47.8%)	28 (54.9%)

LB: Luminal B HER+; HER2+: HER2-enriched; TN: triple-negative.

**Table 2:** Allelic, genotypic, and haplotype frequencies of *FOXP3* polymorphisms in total BC sample and in aggressive subtypes.

	Genotype	Controls (*n* = 300)	Total BC (*n* = 117)	LB (*n* = 37)	HER2+ (*n* = 26)	TN (*n* = 54)
g.10403A>G (rs2232365)	AA	47 (15.7%)	26 (22.2%)	9 (24.3%)	5 (19.2%)	12 (22.2%)
	AG	147 (49.0%)	54 (46.2%)	15 (40.6%)	13 (50.0%)	26 (48.2%)
	GG	106 (35.3%)	37 (31.6%)	13 (35.1%)	8 (30.8%)	16 (29.6%)
	Allele A	40.2%	45.3%	44.6%	44.2%	46.3%
	Allele G	59.8%	54.7%	55.4%	55.8%	53.7%

g.8048A>C (rs3761548)	AA	41 (13.7%)	14 (12%)	7 (18.9%)	4 (15.4%)	3 (5.6%)
	AC	132 (44.0%)	48 (41%)	16 (43.3%)	10 (38.5%)	22 (40.7%)
	CC	127 (42.3%)	55 (47%)	14 (37.8%)	12 (46.1%)	29 (53.7%)
	Allele A	35.7%	32.5%	40.5%	34.6%	25.9%
	Allele C	64.3%	67.5%	59.5%	65.4%	74.1%

Haplotypes	AA	7 (1.2%)	3 (1.3%)	2 (2.7%)	1 (1.9%)	0 (0%)
	AC	235 (39.2%)	104 (44.4%)	31 (41.9%)	22 (42.3%)	51 (47.2%)
	GA	208 (34.6%)	73 (31.2%)	28 (37.8%)	17 (32.7%)	28 (25.9%)
	GC	150 (25%)	54 (23.1%)	13 (17.6%)	12 (23.1%)	29 (26.9%)

LB: Luminal B HER2+; HER2+: HER2-enriched; TN: triple negative.

**Table 3:** Correlation analysis of *FOXP3* polymorphisms in relation to prognostic parameters in total BC sample and aggressive subtypes.

	Clinical outcomes	Total BC *p* (tau)	Breast cancer subtypes [*p* (tau)]		
			LB	HER2+	TN
g.10403A>G	TNM staging	0.173 (*τ* = 0.11)	0.608 (*τ* = 0.08)	0.835 (*τ* = −0.04)	0.032 (*τ* = 0.23)^∗^
	Tumor size	0.633 (*τ* = −0.04)	0.885 (*τ* = 0.02)	0.778 (*τ* = 0.05)	0.422 (*τ* = −0.10)
	Ki-67	0.270 (*τ* = 0,11)	0.837 (*τ* = 0.04)	0.019 (*τ* = 0.47)^∗^	0.536 (*τ* = 0.08)
	Histological grade	0.268 (*τ* = −0.10)	0.846 (*τ* = −0.03)	0.061(*τ* = −0.36)	0.909 (*τ* = −0.02)
	LP commitment	0.298 (*τ* = 0.09)	0.337 (*τ* = 0.15)	0.175(*τ* = 0.255)	0.934 (*τ* = −0.01)

g.8048A>C	TNM staging	0.966 (*τ* = −0.003)	0.894 (*τ* = 0.02)	0.167 (*τ* = 0.23)	0.084(*τ* = −0.23)
	Tumor size	0.166 (*τ* = 0.10)	0.403 (*τ* = 0.11)	0.912 (*τ* = −0.02)	0.419(*τ* = 0.10)
	Ki-67	0.557 (*τ* = −0.06)	0.28 (*τ* = −0.21)	0.018 (*τ* = −0.47)^∗^	0.708 (*τ* = 0.05)
	Histological grade	0.135 (*τ* = −0.15)	0.754 (*τ* = 0.06)	0.026 (*τ* = 0.39)^∗^	0.927 (*τ* = 0.01)
	LP commitment	0.895 (*τ* = −0.01)	0.662 (*τ* = −0.07)	0.811(*τ* = −0.05)	0.913 (*τ* = 0.02)

Kendall's tau test; ^∗^value of *p* < 0.05 was considered statistically significant. BC: breast cancer; LB: Luminal B HER2+; HER2+: HER2-enriched; TN: triple-negative; LP: lymph node.

**Table 4:** *FOXP3* haplotypes correlation analysis in relation to prognostic parameters in total BC sample and aggressive subtypes.

	Clinical outcomes	Haplotypes		
		AC	GA	GC
Total BC	TNM staging	0.07 (*τ* = −0.16)	0.885 (*τ* = 0.01)	0.06 (*τ* = 0.18)
	Histological grade	0.415 (*τ* = 0.07)	0.07 (*τ* = −0.17)	0.377 (*τ* = 0.08)
	Tumor size	0.572 (*τ* = 0.05)	0.157 (*τ* = −0.12)	0.853 (*τ* = 0.01)
	Ki-67 index	0.374 (*τ* = −0.09)	0.517 (*τ* = 0.06)	0.809 (*τ* = 0.03)
	LP commitment	0.309 (*τ* = −0.09)	0.825 (*τ* = 0.02)	0.443 (*τ* = 0.07)

LB	TNM staging	0.256 (*τ* = −0.18)	0.656 (*τ* = −0.07)	0.330 (*τ* = 0.17)
	Histological grade	0.771 (*τ* = −0.05)	0.597 (*τ* = −0.09)	0.392 (*τ* = 0.13)
	Tumor size	0.836 (*τ* = −0.03)	0.491 (*τ* = −0.10)	0.257 (*τ* = 0.17)
	Ki-67 index	0.970 (*τ* = −0.01)	0.118 (*τ* = 0.25)	0.177 (*τ* = −0.26)
	LP commitment	0.135 (*τ* = −0.23)	0.972 (*τ* = 0.01)	0.295 (*τ* = 0.17)

HER2+	TNM staging	0.875 (*τ* = 0.03)	0.186 (*τ* = −0.24)	0.06 (*τ* = 0.34)
	Histological grade	0.104 (*τ* = 0.31)	0.009 (*τ* = −0.15)^∗^	0.968 (*τ* = 0.01)
	Tumor size	0.491 (*τ* = −0.13)	0.811 (*τ* = −0.04)	0.976 (*τ* = 0.01)
	Ki-67 index	0.005 (*τ* = −0.54)^∗^	0.036 (*τ* = 0.43)^∗^	0.876(*τ* = −0.04)
	LP commitment	0.756 (*τ* = −0.06)	0.373 (*τ* = 0.17)	0.955 (*τ* = −0.01)

TN	TNM staging	0.027 (*τ* = −0.29)^∗^	0.044 (*τ* = 0.29)^∗^	0.591 (*τ* = 0.08)
	Histological grade	0.861 (*τ* = 0.02)	0.705 (*τ* = −0.06)	0.750 (*τ* = 0.04)
	Tumor size	0.315 (*τ* = 0.13)	0.419 (*τ* = −0.10)	0.502 (*τ* = −0.09)
	Ki-67 index	0.632 (*τ* = −0.06)	0.708 (*τ* = −0.05)	0.533 (*τ* = 0.09)
	LP commitment	0.883 (*τ* = −0.02)	0.913 (*τ* = −0.02)	0.833 (*τ* = 0.03)

Kendall's tau test; ^∗^value of *p* < 0.05 was considered statistically significant. LP: lymph node.
